# Health-related quality of life in patients newly diagnosed with prostate cancer: CAPLIFE study

**DOI:** 10.1007/s11136-022-03302-z

**Published:** 2022-11-21

**Authors:** Macarena Lozano-Lorca, Rocío Barrios-Rodríguez, Daniel Redondo-Sánchez, José-Manuel Cózar, Miguel Arrabal-Martín, Marta García-Caballos, Inmaculada Salcedo-Bellido, María-José Sánchez, José-Juan Jiménez-Moleón, Rocío Olmedo-Requena

**Affiliations:** 1 Universidad de Granada, Departamento de Medicina Preventiva y Salud Pública, 18016 Granada, Spain; 2 Instituto de Investigación Biosanitaria Ibs.GRANADA, 18014 Granada, Spain; 3 Consortium for Biomedical Research in Epidemiology and Public Health (CIBERESP), 28029 Madrid, Spain; 4 Andalusian School of Public Health (EASP), Campus Universitario de Cartuja, C/Cuesta del Observatorio 4, 18080 Granada, Spain; 5 Urology Department, Virgen de las Nieves University Hospital, 18014 Granada, Spain; 6 Urology Department, San Cecilio University Hospital, 18016 Granada, Spain; 7 Cartuja Primary Health Care Centre, Distrito Sanitario Granada-Metropolitano, 18013 Granada, Spain

**Keywords:** Prostate cancer, Health-related quality of life, Urinary symptomatology, Tumour extension

## Abstract

**Purpose:**

To analyse the Health-Related Quality of Life (HRQoL) at diagnosis of patients with prostate cancer (PCa) according to tumour extension and urinary symptomatology and to explore factors associated with HRQoL.

**Methods:**

408 Controls and 463 PCa cases were included. Eligibility criteria were a new diagnosis of PCa (cases), 40–80 years of age, and residence in the participating hospitals’ coverage area for ≥ 6 months before recruitment. HRQoL was evaluated using the 12-Item Short-Form Health Survey, Mental (MCS) and Physical Component Summaries (PCS), and urinary symptoms with the International Prostate Symptom Score. HRQoL scores for all PCa cases, according to tumour extension and urinary symptoms, were compared with controls. In addition, information about lifestyles and comorbidities was collected and its association with low HRQoL (lower scores) were explored using logistic regression models.

**Results:**

Overall cases had similar PCS score, but lower MCS score than controls. The lowest standardised scores for both PCS and MCS were reached by cases with severe urinary symptoms and a metastatic tumour [mean (SD); PCS: 41.9 (11.5), MCS: 42.3 (10.3)]. Having “below” PCS and MCS scores was associated with the presence of three or more comorbidities in the cases [aOR = 2.86 (1.19–6.84) for PCS and aOR = 3.58 (1.37–9.31) for MCS] and with severe urinary symptomatology [aOR = 4.71 (1.84–12.08) for PCS and aOR = 7.63 (2.70–21.58) for MCS].

**Conclusion:**

The mental dimension of HRQoL at diagnosis of patients with PCa was lower than in controls, especially for cases with severe urinary symptoms and a metastatic tumour. Comorbidities and urinary symptoms were variables associated with the HRQoL of PCa cases.

**Supplementary Information:**

The online version contains supplementary material available at 10.1007/s11136-022-03302-z.

## Plain English summary

Prostate cancer cases have increased in recent years, but mortality rates do not present the same trend. On the contrary, there has been an increase in the number of survivors, and quality of life considered a valuable measurement beyond mere survival. Although the impact of treatments on the quality of life of patients with prostate cancer is well known, this issue is not well explored at the moment of the diagnosis. This study analyses the quality of life at diagnosis of prostate cancer patients, comparing this with a control group of the same base population. Moreover, we evaluated the influence of tumour extension and urinary symptoms and explored other potential factors associated with quality of life in these patients. The results showed that patients with prostate cancer had lower quality of life scores than controls, especially for mental health and in cases with severe symptoms and a metastatic tumour. In addition, lower scores in the quality of life of the cases were associated with the presence of comorbidities and urinary symptoms. Considering the variables associated with HRQoL, evaluating urinary symptoms and comorbidities from the moment of diagnosis could be an important aspect to include in the clinical decision making.

## Introduction

Prostate cancer (PCa) poses a significant public health burden and is a major cause of morbidity and mortality worldwide, being the most common cancer in men in most world regions, excluding non-melanoma skin cancers [1, 2]. In recent years, there has been an increase in the number of cases worldwide [3]. Nevertheless, mortality rates have decreased [2]. Spain has become one of the European countries with the lowest mortality rates (13.2 deaths per 100,000 person-years) [4], and the survival rates indicate that approximately 89% of Spanish patients are still alive 5 years after a PCa diagnosis [5].

A consequence of these epidemiological trends of PCa is the growing interest in Health-Related Quality of Life (HRQoL) as a valuable measurement beyond mere survival [6]. The impact of treatments on HRQoL has been widely studied [7]. In this sense, some studies have reported how the different treatments were associated with distinct patterns of adverse effects [8–11]. Recently, it has been suggested that HRQoL should be integrated into the treatment decision-making process [12, 13]. However, the HRQoL of PCa patients is not only relevant with regard to treatment. It has been shown that the loss of quality of life during the process of diagnosis is one of the most relevant aspects for PCa patients [14, 15].

Previous studies have analysed the HRQoL of PCa patients at the time of diagnosis [16–20]; however*,* we must consider: (i) the results regarding HRQoL were not always similar between studies, and some found that the HRQoL score is similar to that of the general population [16, 17] and others better [18, 19]. These discrepancies may be due to differences in the characteristics of PCa cases—such as symptoms, tumour aggressiveness or extension, sociodemographic variables, or lifestyles—; (ii) only 2 studies have used the SF-12, allowing the differentiation between physical and mental quality of life; and (iii) factors related to HRQoL differ between studies, with most focusing on non-modifiable (age, bone metastases, Gleason score) factors or those that are difficult to modify (comorbidities or region of residence) [16–20]. However, to our knowledge, no study has evaluated potentially modifiable factors associated with HRQoL in newly diagnosed patients [16–20]. Moreover, the impact that symptoms have on HRQoL during diagnosis maybe be a crucial aspect for PCa patients [15], which has not been studied until now. Such a study could provide useful information to formulate individualised interventions to improve HRQoL from the moment of diagnosis, as has been suggested previously [21].

Thus, considering the increase in the number of prevalent and incident cases of PCa, the importance of the assessment of HRQoL of patients during the PCa diagnostic process, the inconsistency between previous studies, and the lack of studies that consider urinary symptoms as a factor that could be associated with HRQoL, the aims of this study were (1) to compare HRQoL, using the Short-Form Health Survey version 2 (SF-12 v2), of PCa cases at diagnosis with controls from the same base population, according to tumour extension and urinary symptoms through the International Prostate Symptom Score; and (2) to explore possible factors associated with HRQoL of these patients.

## Methods

### Study design

The CAPLIFE study is a population-based case–control study whose main objective was to investigate the association between lifestyles and PCa risk. This study was carried out at two main University Hospitals in Granada (Spain): Virgen de las Nieves and Clínico San Cecilio University Hospitals and their catchment areas. Participants were invited to participate from May 2017 to March 2020. The main characteristics of the CAPLIFE study have been described elsewhere [22, 23].

Ethical Approval for this study was provided by the Ethics Committee for Biomedical Research of Andalusia in March 2017. All men included were fully informed regarding the study and signed written informed consent before their voluntary participation. Confidentiality of data was secured, removing personal identifiers in the dataset.

### Participants

Criteria for inclusion of cases: (1) new diagnosis of PCa with histological confirmation before receiving treatment (International Classification of Diseases and Related Health Problems 10th Revision [ICD-10]: C61 [24]), (2) 40–80 years of age, and (3) residence in the coverage area of the reference hospitals for 6 months or more prior to recruitment. Controls were selected using the same selection criteria as the cases except for the diagnosis of PCa; therefore, the only difference between cases and controls was the disease (PCa).

Cases were recruited and invited to participate in the urology services of both hospitals through the listings of Pathological Anatomy Service. Controls were chosen at random from the patient lists of general practitioners of 16 primary healthcare centres belonging to the Granada-Metropolitan Health District. For the recruitment of controls, the age at diagnosis of PCa (± 5 years) based on the information obtained from the Granada Cancer Registry was considered. Controls were selected by density sampling over time, which allowed greater comparability of cases and controls. Trained interviewers contacted them to make an appointment for the face-to-face interviews.

### Data collection

Information was collected using a structured computerised questionnaire and participants’ medical history.

#### Health-related quality of life (HRQoL)

The SF-12 v2 was used to evaluate HRQoL during the month before the interview. This validated instrument contains 12 questions with 3- or 5-point Likert scales resulting in 8 dimensions (each dimension was built with 1 or 2 questions): Physical Functioning, Role Physical, Bodily Pain, General Health, Vitality, Social Functioning, Emotional Role, and Mental Health [25]. Additionally, two component summaries are calculated from these eight dimensions: Physical Component Summary (PCS) and Mental Component Summary (MCS). Standards-based scores were obtained following the instructions detailed in the manual of the original version of the questionnaire [26]. For standardisation of the 8 dimensions, we used the means and standard deviation. The weighted dimensions were summed to calculate the summary components (PCS and MCS). Means, standard deviations, and the weights established for the Spanish population were used [27]. Finally, both dimensions and summary components were normalised. Higher scores indicate better quality of life. The Cronbach alpha for SF-12 v2 in our sample was 0.88, which indicates acceptable internal consistency reliability.

#### Measurement of clinical characteristics

The tumour extension was determined according to the European Association of Urology risk groups for biochemical recurrence: localised, locally advanced, and metastatic PCa [28]. The International Society of Urological Pathology was used to determine low (1–2) and high aggressiveness (3–5) [29, 30].

The original International Prostate Symptom Score [31], validated in the Spanish population, was selected to evaluate urinary symptoms [32]. This was based on the answers to seven questions (incomplete emptying, frequency, intermittency, urgency, weak stream, straining, and nocturia) and referred to the month before the interview. Each question is scored from 0 to 5; the total score is obtained by adding the score of the items and ranges from 0 to 35 points (a higher score indicates more severe symptoms). A Cronbach's α for the International Prostate Symptom Score items of 0.86 was found for our sample. Participants were classified as follows: (i) without (0 points), (ii) mild (1–7 points), iii) moderate (8–19 points), and iv) severe urinary symptoms (20–35 points). We added the category “without urinary symptoms” to differentiate between those with no symptoms and those with mild symptoms.

#### Sociodemographic data and personal history

Information was also collected on sociodemographic data (age, education, marital status, and employment status), Body Mass Index (BMI), first-degree family history of PCa, lifestyle factors (smoking status, sedentary, and Mediterranean diet pattern), and comorbidities.

To evaluate comorbidities, subjects were asked if they had been diagnosed with the following diseases: diabetes mellitus (type 1 or 2), cardiovascular diseases (stroke, angina pectoris, and/or acute myocardial infarction), respiratory diseases (asthma and/or chronic obstructive pulmonary disease), hypertension, mental illnesses (anxiety and/or depression), and other previous cancers. From this, two categories were created following what was done by Porreca A et al*.* in the Pros-IT CNR study [18]: (i) presence of two or fewer comorbidities, and (ii) presence of three or more comorbidities.

The International Physical Activity Questionnaire Short-Form (IPAQ-SF), validated for the Spanish population [33], was used to collect information about physical activity and sedentariness, referring to the week prior to the interview. The level of sedentariness was measured (h/day) and categorised in terciles based on the control group cut-off points (i) tertile 1 (T1): ≤ 6 h/day); (ii) tertile 2 (T2): > 6 −  ≤ 9 h/day; and (iii) tertile 3 (T3) > 9 h/day.

In addition, to determine the adherence to the Mediterranean diet pattern, subjects were provided with a semi-quantitative validated Food Frequency Questionnaire (FFQ) [34]. This questionnaire included 134 foods, specifying the portion size for each food, and referred to the 12 months before the interview. Intakes of less than 800 kcal/day and more than 4000 kcal/day were considered implausible extreme energy intakes [35]. Mediterranean diet pattern adherence was measured by MedDietScore [36]. The total score is obtained by adding the score of the 11 items and ranged from 0 (minimum adherence) to 55 (maximum adherence).

### Statistical analysis

In the descriptive analysis, the mean and standard deviation (SD) of the continuous quantitative variables and the distribution of absolute and relative frequencies for the qualitative variables were calculated. Student’s *t* tests, one-way ANOVA, or Chi-squared test were used according to the nature of the variables.

The HRQoL scores of the cases were compared with scores of the dimensions and components of the SF-12v2 of the control group. For this, both groups were categorised by age groups (40–54, 55–64, 65–74, 75–80 years). The effect size of the differences between PCa cases and controls scores was determined using Cohen's *d.* It was calculated as the mean difference divided by the standard deviation of the control group (it takes value 10 being normalised scores). An effect size of 0.2, 0.5, and 0.8 was considered as small, medium, or large, respectively [37].

To identify factors associated with HRQoL of cases, they were classified as “the same as or better” or “below” PCS and MCS scores than controls according to age group. Multivariable logistic regression models were used to estimate odds ratios (OR) and 95% confidence intervals (95% CI). Only variables with *p* values < 0.20 in the crude analysis were included in these multivariate models.

All statistical tests were two sided and statistical significance was set at *p* < 0.05. Statistical analyses were performed using the statistical program Stata v.15 (Stata Corp., 2017, College Station, TX, USA). Interactive radar plots showing HRQoL by age group are available online as a web application using R software version 3.6.1 and the packages shiny (v.1.4.0.2) and radarchart (v.0.3.1).

## Results

**Fig. 1:**
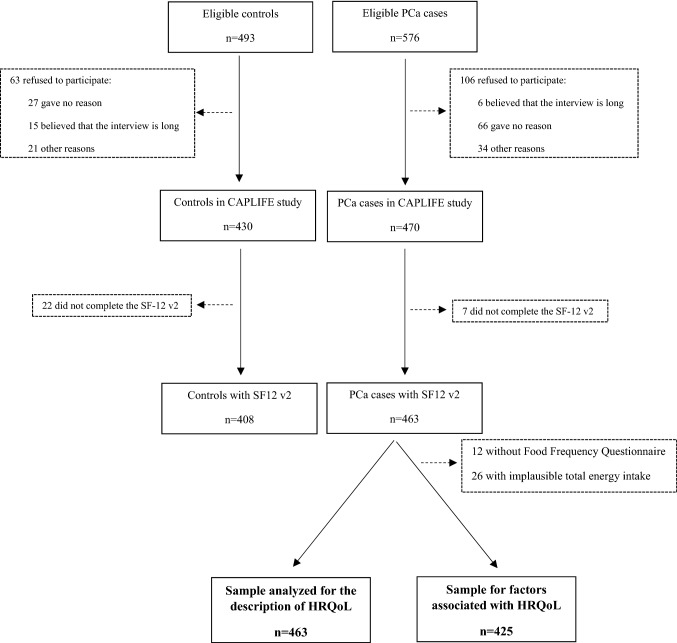
Flow-chart of participants in the CAPLIFE study

A total of 493 controls and 576 patients with confirmed PCa were invited to participate in the CAPLIFE study. Of these, 430 controls and 470 cases accepted participation in the study. Finally, 408 controls (94.9%) and 463 cases (98.5%) completed the SF-12 v2 (Fig. 1).

### Prostate cancer cases and controls characteristics

PCa cases were slightly older, 67.6 years (SD 7.5) vs. 66.0 (SD 7.7), and had a first-degree family history of PCa more frequently (21.9% vs. 10.5%) than the controls. Only 6.7% of cases and 6.6% of the controls had 3 or more comorbidities. Severe urinary symptoms were presented only in 7.1% of the PCa patients, while 27.4% had no urinary symptoms and 38.9% had mild urinary symptoms. According to the tumour extension, metastatic PCa cases were more sedentary, had severe symptoms, and a more aggressive tumour than patients with localised tumours (Table 1).

**Table 1:** Characteristics of controls and overall prostate cancer (PCa) cases and according to tumour extension in the CAPLIFE study

	Controls	Overall PCa cases	*p* value	Localised PCa cases	Locally advanced PCa cases	Metastatic PCa cases	*p* value
	*n* (%)	*n* (%)		*n* (%)	*n* (%)	*n* (%)	
Total	408 (100.0)	463 (100.0)		401 (86.6)	39 (8.4)	23 (5.0)	
Age (years), mean (SD)	66.0 (7.7)	67.6 (7.5)	0.002	67.4 (7.5)	67.7 (7.4)	70.8 (6.8)	0.076
*Age (years), n (%)*			0.005				0.460
40−54	38 (9.3)	28 (6.1)		26 (6.5)	1 (2.6)	1 (4.4)	
55−64	146 (35.8)	134 (28.9)		119 (29.7)	12 (30.8)	3 (13.0)	
65−74	174 (42.7)	214 (46.2)		184 (45.9)	16 (41.0)	14 (60.9)	
75−80	50 (12.2)	87 (18.8)		72 (18.0)	10 (25.6)	5 (21.7)	
*Education, n (%)*			0.230				0.629
Primary	115 (28.2)	142 (30.7)		126 (31.4)	9 (25.6)	8 (30.4)	
Secondary	203 (49.7)	240 (51.8)		208 (51.9)	22 (52.3)	10 (43.5)	
University	90 (22.1)	81 (17.5)		67 (16.7)	8 (22.1)	6 (26.1)	
*Marital status, n (%)*			0.820				0.804
Married	336 (82.3)	384 (82.9)		333 (83.0)	33 (84.6)	18 (78.3)	
Not married	72 (17.7)	79 (17.1)		68 (17.0)	6 (15.4)	5 (21.7)	
*Employment status, n (%)*			0.628				0.300
Retired	273 (66.9)	315 (68.0)		267 (66.6)	28 (71.8)	20 (87.0)	
Still working	109 (26.7)	113 (24.4)		102 (25.4)	8 (20.5)	3 (13.0)	
Unemployed	26 (6.4)	35 (7.6)		32 (8.0)	3 (7.7)	0 (0.0)	
*First-degree family history of PCa, n (%)*			< 0.001				0.977
No	265 (89.5)	361 (78.1)		312 (78.0)	31 (79.5)	18 (78.3)	
Yes	43 (10.5)	101 (21.9)		88 (22.0)	8 (20.5)	5 (21.7)	
Unknown	-	1		1	–	–	
*Comorbilities, n (%)*			0.971				0.37.0
0–2	380 (93.4)	432 (93.3)		372 (92.8)	37 (94.9)	23 (100.0)	
≥ 3	27 (6.6)	31 (6.7)		29 (7.2)	2 (5.1)	-	
*BMI, n (%)*			0.558				0.259
Normal weight	72 (17.6)	95 (20.5)		79 (19.7)	7 (18.0)	9 (39.1)	
Overweight	216 (52.9)	238 (51.4)		209 (52.1)	20 (51.3)	9 (39.1)	
Obesity	120 (29.4)	130 (28.1)		113 (28.2)	12 (30.8)	5 (21.7)	
*Smoking status, n (%)*			0.789				0.215
Never smoker	105 (25.7)	117 (25.3)		95 (23.7)	13 (33.3)	9 (39.1)	
Former smoker	226 (55.4)	250 (54.0)		221 (55.1)	17 (43.6)	12 (52.2)	
Current smoker	77 (18.9)	96 (20.7)		85 (21.2)	9 (20.1)	2 (8.7)	
*Sedentary, n (%)*			0.703				0.027
T1 (≤ 6 h/day)	186 (45.8)	199 (43.0)		181 (45.1)	13 (33.3)	5 (21.7)	
T2 (6–9 h/day)	118 (29.1)	142 (30.7)		121 (30.2)	15 (38.5)	6 (26.1)	
T3 (> 9 h/day)	102 (25.1)	122 (26.3)		99 (24.7)	11 (28.1)	12 (52.2)	
Missing	2	–		–	–	–	
Mediterranean diet pattern, mean (SD)	35.0 (4.0)	34.9 (3.8)	0.810	35.0 (3.8)	33.7 (3.9)	35.0 (3.2)	0.147
*Urinary symptoms, n (%)*			–				< 0.001
Without	–	127 (27.4)		103 (25.7)	14 (35.9)	10 (43.5)	
Mild	–	180 (38.9)		161 (40.2)	15 (38.5)	4 (17.4)	
Moderate	–	123 (26.6)		114 (28.4)	7 (18.0)	2 (8.7)	
Severe	–	33 (7.1)		23 (5.7)	3 (7.7)	7 (30.4)	
*Tumour aggressiveness*^*a*^*, n (%)*			–				< 0.001
Low (ISUP 1–2)	–	353 (76.4)		327 (81.6)	24 (61.5)	2 (9.1)	
High (ISUP 3–5)	–	109 (23.6)		74 (18.4)	15 (38.5)	20 (90.9)	

*BMI* body mass index, *SD* standard deviation, *T* Tercile

^a^One subject could not be categorised using ISUP classification as it was a neuroendocrine carcinoma

### Scores of physical and mental component summaries

Overall cases had very similar PCS scores but lower MCS scores than controls [53.7 (SD 8.1) vs. 50.8 (SD 9.7)] (Table 2). The most impaired scores were obtained by those cases with a metastatic PCa tumour and severe urinary symptoms [PCS: 41.9 (11.5); MCS: 42.3 (10.3)], with a large effect size for PCS and MCS (0.81 and 1.15, respectively), compared to the controls.

**Table 2:** Component summaries of short-form health survey version 2 (SF-12 v2) score of controls and prostate cancer (PCa) cases according to European association of urology (EAU) risk groups and urinary symptoms in the CAPLIFE study

SF-12 v2 score	Controls	Overall PCa cases			Localised PCa cases			Locally advanced PCa cases			Metastatic PCa cases		
Total, *n* (%)	408 (100.0)	463 (100.0)			354 (76.5)			86 (18.5)			23 (5.0)		
	Mean (SD)	Mean (SD)	Diff.	Effect size	Mean (SD)	Diff.	Effect size	Mean (SD)	Diff.	Effect size	Mean (SD)	Diff.	Effect size
PCS	49.9 (7.6)	50.2 (8.2)	0.4	0.04	50.5 (7.9)	0.7	0.07	50.1 (8.6)	0.2	0.11	44.4 (10.2)	−5.1	0.51
Urinary symptoms													
Without		51.3 (7.0)	1.6	0.16	51.5 (7.0)	1.8	0.18	51.9 (6.5)	1.7	0.17	48.3 (7.5)	−0.9	0.09
Mild		50.5 (7.5)	0.7	0.07	50.9 (7.2)	1.0	0.10	49.6 (7.4)	−0.1	−0.01	39.9 (10.6)	−9.3	0.93
Moderate		50.2 (8.3)	0.4	0.04	50.3 (7.8)	0.4	0.04	50.8 (12.8)	1.1	0.11	42.2 (19.6)	−7.2	0.72
Severe		43.7 (12.6)	−6.2	0.62	44.3 (13.3)	−5.5	0.55	42.9 (12.6)	−6.6	0.66	41.9 (11.5)	−8.1	0.81
MCS	53.7 (8.1)	50.8 (9.7)	−3.3	0.33	50.8 (9.8)	−3.2	0.32	51.7 (8.2)	−2.5	0.25	48.5 (9.9)	−6.1	0.61
Urinary symptoms													
Without		53.1 (8.5)	−1.2	0.12	53.0 (9.0)	−1.3	0.13	54.0 (5.3)	0.0	0.00	52.7 (7.4)	−2.0	0.20
Mild		50.5 (9.6)	−3.5	0.35	50.1 (9.9)	−3.9	0.39	53.7 (6.3)	−0.5	0.05	52.5 (9.7)	−2.5	0.25
Moderate		50.0 (10.2)	−4.0	0.40	50.5 (9.9)	−3.4	0.34	44.6 (13.3)	−9.6	0.96	40.7 (11.0)	−14.7	1.47
Severe		46.8 (10.4)	−7.4	0.74	48.0 (10.9)	−6.2	0.62	48.3 (4.2)	−7.3	0.73	42.3 (10.3)	−11.5	1.15

*Diff.* Difference, *MCS* Mental Component Summary, *PCS* Physical Component Summary, *SD* standard deviation

Each man was compared with the specific group of controls of the same age range. For men between 40–44 years: PCS: 50.7, MCS: 49.3; 45–54 years: PCS: 49.7, MCS: 52.3; 55–64 years: PCS: 51.1, MCS: 52.2; 65–74 years: PCS: 49.0, MCS: 54.7; and ≥ 75 years: PCS: 49.8, MCS: 56.1

Characteristics of PCa cases categorised as the same/better or below PCS and MCS scores than controls are available in Supplementary Table 1. Overall, 29.4% of PCa had a lower score in PCS, and 52.9% had a lower score in MCS at the moment of diagnosis.

### Scores of the eight dimensions of the SF-12 v2

**Fig. 2:**
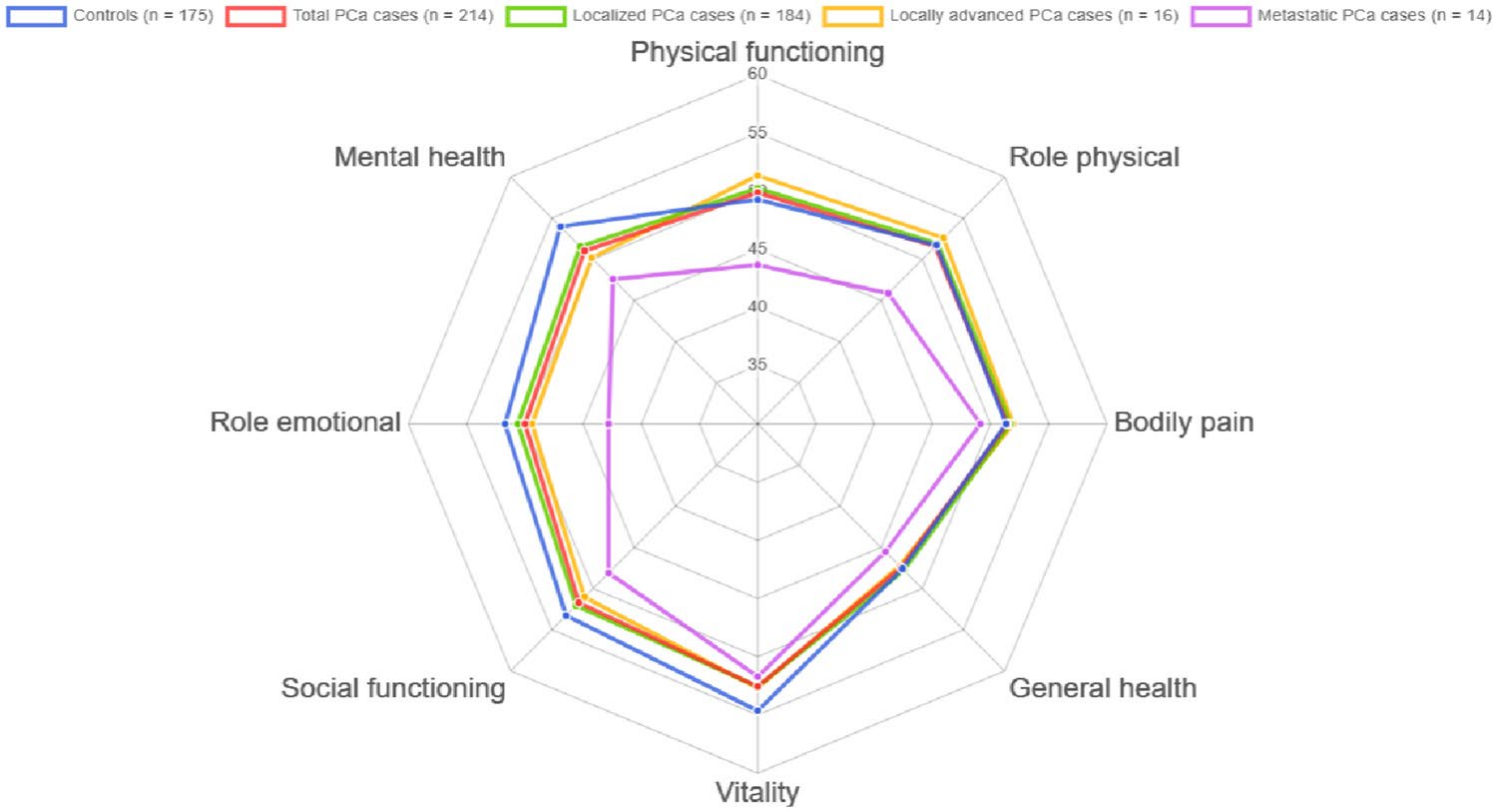
Scores for the 8 dimensions of the Short-Form Health Survey version 2 (SF-12 v2) in prostate cancer (PCa) cases and controls aged 65–74 years (the largest group)

The comparison of the scores for the 8 dimensions of SF-12 v2, for all PCa cases and stratified by tumour extension, and considering the age group is shown in Online Resource 1 (https://dredondo.shinyapps.io/caplife/). Mental Health was impaired in PCa cases with a small–medium effect size for all age ranges. According to the tumour extension, in the group of 65–74 and 75–80 years, metastatic PCa cases had lower scores than localised cases and locally advanced cases in all dimensions. The same occurs in the rest of the age groups (40–54 and 55–64 years), except for the Mental Health dimension. Figure 2 shows only the comparison of the age group with more PCa cases: 65–74 years.

### Factors associated with “below” scores of physical and mental component summaries

Table 3 shows the factors associated with having below scores in PCS and MCS compared with the control group. Comorbidities and urinary symptoms were the only variables associated with both PCS and MCS scores. Cases with three or more comorbidities had higher odds of having below scores for both PCS [aOR = 2.86 (95% CI 1.19–6.84)] and MCS [aOR = 3.58 (95% CI 1.37–9.31)]. In the same way, severe urinary symptomology was associated with a below score for PCS [aOR = 4.71 (95% CI 1.84–12.08)] and MCS [aOR = 7.63 (95% CI 2.70–21.58)]. Additionally, there was a significant dose–response relationship between hours a day sitting and having a below PCS score: PCa cases who spent > 6–9 h and ≥ 9 h a day sitting had 2.65 (95% CI 1.48–4.75) and 3.88 (95% CI 2.13–7.06) times more odds of having below PCS score than those who spent ≤ 6 h, respectively. Regarding MCS, every one-unit increase in the score of adherence to a Mediterranean dietary pattern was associated with having the same or better MCS score than controls, with an aOR = 0.94 (95% CI 0.89–0.99). These models explained 14.3% and 7.8% of the variance of PCS and MCS, respectively.

**Table 3:** Multivariable analysis to explore the factors associated with below physical (PCS) and mental (MCS) component summary scores of the short-form health survey version (SF-12 v2) in cases (*n* = 425)

	Below PCS^a^ score	Below MCS^b^ score
	aOR (95% CI)	aOR (95% CI)
Age (5 years increase)	1.09 (0.88–1.36)	–
*Education*		
University	Reference	Reference
Secondary	0.91 (0.46–1.80)	0.92 (0.53–1.60)
Primary	1.69 (0.80–3.58)	1.31 (0.71–2.42)
*Employment status*		
Retired	1.00	–
Still working	0.70 (0.31–1.57)	–
Unemployed	1.41 (0.52–3.58)	–
*First-degree family history of PCa*		
No	Reference	–
Yes	0.97 (0.54–1.74)	–
*Comorbidities*		
0–2	Reference	Reference
≥ 3	2.86 (1.19–6.84)	3.58 (1.37–9.31)
*BMI*		
Normal weight	Reference	-
Overweight	0.60 (0.32–1.14)	-
Obesity	1.08 (0.55–2.12)	-
*Smoking status*		
Never smoker	–	Reference
Former smoker	–	0.63 (0.39–1.01)
Current smoker	–	1.03 (0.56–1.90)
*Sedentary lifestyle*		
Tercile 1 (≤ 6 h/day)	Reference	–
Tercile 2 (6–9 h/day)	2.65 (1.48–4.75)	–
Tercile 3 (≥ 9 h/day)	3.88 (2.13–7.06)	–
Mediterranean diet pattern (1-unit increase)	–	0.94 (0.89–0.99)
*EAU risk groups*		
Localised PCa cases	Reference	
Locally advanced PCa cases	1.36 (0.59–3.11)	
Metastatic PCa cases	2.42 (0.81–7.20)	
*Urinary symptoms*		
Without	Reference	Reference
Mild	2.23 (1.21–4.10)	1.52 (0.92–2.49)
Moderate	1.61 (0.81–3.19)	1.81 (1.06–3.11)
Severe	4.71 (1.84–12.08)	7.63 (2.70–21.58)

*aOR* adjusted odds ratio, *BMI* body mass index, *EAU* European Association of Urology, *PCa* Prostate Cancer

^a^Variables included in the model: age, education, employment status, first-degree family history of PCa, comorbidities, BMI, sedentary lifestyle, European Association of Urology risk groups and urinary symptoms

^b^Variables included in the model: education, comorbidities, smoking status, Mediterranean diet pattern and urinary symptoms

## Discussion

Our results indicated that there was a higher percentage of PCa cases with a below MCS score than with a below PCS score compared to the control group and these differences were higher with greater tumour extension and severe urinary symptoms. Regarding the factors associated with poor HRQoL, more severe urinary symptoms and the presence of three or more comorbidities were associated with both component summaries. In addition, sedentariness had a negative impact on PCS, while less adherence to the Mediterranean diet was associated with a below MCS.

In general, PCa cases had very similar PCS scores to controls, consistent with previous studies [18, 19]. However, there was more discrepancy with respect to MCS. We found that PCa patients had more impaired MCS scores than controls. Lane et al*.* [17] and Harju et al*.* [19] found similar mental health scores, comparing the patients' HRQoL to UK and Finnish normative data, respectively. These results and the differences with ours may be due to the low aggressiveness of PCa cases analysed in these studies: Lane et al*.* analysed only localised PCa, and in the study of Harju et al*.* more than one‐third of the patients initially received non-invasive care. This explanation is supported by the finding that tumour extension had implications for the HRQoL of our patients, with lower scores for locally advanced and metastatic PCa cases, especially for the MCS. Other studies have also found lower scores in HRQoL components in patients with more advanced PCa at diagnosis [16, 18].

In the CAPLIFE study, comorbidity and urinary symptoms were key variables for the HRQoL of PCa patients. The presence of three or more comorbidities was associated with worse HRQoL, both physical and mental. Similar findings have been found in previous studies [18, 19]. In addition, a cohort study observed this effect of comorbidity on PCS from pre-treatment to 36 months [38]. Regarding urinary symptomatology, we added the category “without urinary symptoms” to differentiate between those who had no symptoms and those who had mild symptoms. In our study, those patients with severe symptoms had the most impaired scores, both in PCS and MCS, especially in metastatic cases. Previous studies have suggested paying more attention to identifying and supporting long-term survivors who experience multiple symptoms [39, 40].

Regarding other factors related to HRQoL, we found that being sedentary was associated with below PCS scores and also observed a positive trend between tumour extension and this dimension. Different results have been found in previous studies. Older age, the presence of bone metastases, and higher Gleason score were the factors associated with worse general HRQoL scores in the study of Bergius et al*.* [16]. This study used a different HRQoL questionnaire, EORTC QLQ-C30, and only included clinical factors in the models. On the other hand, the Pros-IT CNR study [18] found older age, obesity, having comorbidities, and a Gleason score ≥ 8 to be associated with worse PCS score. In this case, the discrepancies could be due to differences in: (1) the included variables: sedentary, first-degree family history of PCa, urinary symptoms, tumour extension, and diet were not evaluated in the Pros-IT CNR study, and (2) the analytical model: we evaluated factors associated with having worse HRQoL score and not the changes in the means of dimensions as done in the Pros-IT CNR study. With our approximation, the model explained 14.5% of the variance in PCS. These results could have practical implications, as with evaluation over time and intervention in modifiable variables such as sedentariness, urinary symptoms, and comorbidity, the PCS scores of PCa cases could approach to those of the control group.

In addition, we also found that less adherence to the Mediterranean diet pattern was related to a below score in MCS. To the best of our knowledge, no previous study has analysed this variable in relation to HRQoL in PCa patients. Considering that previous studies have shown that the decrease in mental health is maintained as the disease evolves [41], the integration of mental care from the beginning, especially in people with other diseases, could help close the gaps found in the follow-up care of PCa survivorships [42].

Some strengths of the present study are: (i) to our knowledge, it is the first study to evaluate the influence of urinary symptoms on HRQoL in newly diagnosed PCa patients. International Prostate Symptom Score was used to evaluate symptoms and SF-12 v2 for HRQoL. Although there are multiple generic and disease-specific HRQoL instruments, none of them has emerged as a gold standard [43]. Specific questionnaires such as Functional Assessment of Cancer Therapy-Prostate (FACT-P), Expanded Prostate Cancer Index Composite (EPIC), or University of California-Los Angeles Prostate Cancer Index (UCLA-PCI) are especially useful for patients already under treatment [44–46]. It should be noted that SF-12 v2 has been recommended specifically in PCa patients for its psychometric properties and shortness [47]; (ii) PCS and MCS scores of PCa cases were compared to scores in a control group, belonging to the same area of residence and with very similar sociodemographic characteristics. Previous studies carried out the comparison with a reference population, whose sociodemographic characteristics could differ [19, 48]; (iii) a sensitivity analysis was carried out, comparing the HRQoL scores of our cases not only with the controls but also with the published for Spanish reference population, obtaining similar results (data not shown); (iv) our study included patients with diverse tumour extension, aggressiveness, and urinary symptomatology, which helps to characterise the entire spectrum of the disease; and (v) we evaluated the role of potentially modifiable factors associated with HRQoL such as sedentariness or diet, which can be integrated into the supportive care of PCa patients.

Several limitations should be noted. First, we have not used a disease-specific instrument to evaluate HRQoL. However, this fact should not affect the results as PCa-specific questionnaires aimed to establish the impact of treatments, and our patients were not yet being treated. Second, our results may have been limited by the small sample size and lack of statistical power to detect significant associations. Nevertheless, it was enough to find clinically relevant results, particularly in the effect of urinary symptoms and comorbidity on HRQoL. In addition, although we have analysed several potential factors associated with HRQoL in PCa patients, we cannot rule out the influence of other variables. Finally, the controls were sampled via healthcare centres. In addition to being sampled from centres covering the entire province in urban and rural areas, the participants were randomly selected from the list of family physicians, who care for people with and without pathologies; and controls were possible because the Spanish National Health System has universal access, with a family physician assigned to each user (99.6% of the population of Andalusia is covered by the public health system). The invitation by family physicians facilitated a good participation rate (87.2% in controls; 81.6% in cases).

## Conclusions

In conclusion, this study suggests that PCa cases had similar PCS and below MCS scores compared to the control group, and the differences are more pronounced when the cases present severe urinary symptoms and higher tumour extension. Considering the variables associated with the HRQoL of PCa cases, evaluating urinary symptoms and comorbidities from diagnosis could be an important aspect of clinical decision making.

## Supplementary Information

Below is the link to the electronic supplementary material.

Supplementary file1 (DOCX 25 KB)

## Data Availability

Data are available on request due to privacy/ethical restrictions.
