# Supplementary material for: Health-related quality of life in patients newly diagnosed with prostate cancer: CAPLIFE study
Source: Qual Life Res. 2022 Nov 21;32(4):977–88. doi: 10.1007/s11136-022-03302-z (PMC10063519; doi:10.1007/s11136-022-03302-z)
Supplement: Supplementary file 1 — Supplementary file1 (DOCX 25 KB) [file 11136_2022_3302_MOESM1_ESM.docx]

**Health-Related Quality of Life in patients newly diagnosed with prostate cancer: CAPLIFE study**

**Journal name:** Quality of Life Research

Macarena Lozano-Lorca, Rocío Barrios-Rodríguez, Daniel Redondo-Sánchez, José-Manuel Cózar, Miguel Arrabal-Martín, Marta García-Caballos, Inmaculada Salcedo-Bellido, María-José Sánchez, José-Juan Jiménez-Moleón, Rocío Olmedo-Requena^*^

*Correspondence: Rocío Olmedo-Requena. Department of Preventive Medicine and Public Health. School of Medicine. University of Granada. Avda. de la Investigación, 11, Building A, 8th Floor, Office 2. CP 18016, Granada, Spain. E-mail: [rocioolmedo@ugr.es](mailto:rocioolmedo@ugr.es). Phone: (34)-958 243 543.

| **Supplementary Table 1.** Characteristics of PCa cases according to the Physical and Mental Component Summary scores of the SF-12 v2 categorized as “the same as or better” and “below” than the control group. | | | | | | |
| --- | --- | --- | --- | --- | --- | --- |
|  | **PCS score** | |  | **MCS score** | | |
|  | **Same or better** | **Below** |  | **Same or better** | **Below** |  |
|  | n (%) | n (%) | ***p value*** | n (%) | n (%) | ***p value*** |
| **Total** | 327 (70.6) | 136 (29.4) |  | 218 (47.1) | 245 (52.9) |  |
| **Age (years), mean (SD)** | 66.9 (7.6) | 69.2 (7.1) | 0.003 | 67.8 (7.4) | 67.4 (7.6) | 0.556 |
| **Age (years), n (%)** |  |  | 0.001 |  |  | 0.589 |
| 40−54 | 27 (8.3) | 1 (0.7) |  | 10 (4.6) | 18 (7.3) |  |
| 55−64 | 87 (26.6) | 47 (34.6) |  | 67 (30.7) | 67 (27.4) |  |
| 65−74 | 159 (48.6) | 55 (40.4) |  | 100 (45.9) | 114 (46.5) |  |
| 75−80 | 54 (16.5) | 33 (24.3) |  | 41 (18.8) | 46 (18.8) |  |
| **Education, n (%)** |  |  | 0.001 |  |  | 0.080 |
| Primary | 83 (25.4) | 59 (43.4) |  | 56 (25.7) | 86 (35.1) |  |
| Secundary | 183 (56.0) | 57 (41.9) |  | 123 (56.4) | 1177 (47.8) |  |
| Universitary | 61 (18.6) | 20 (14.7) |  | 39 (17.9) | 42 (17.1) |  |
| **Marital status, n (%)** |  |  | 0.448 |  |  | 0.767 |
| Married | 274 (83.8) | 110 (80.9) |  | 182 (83.5) | 161 (82.5) |  |
| Not married | 53 (16.2) | 26 (19.1) |  | 36 (16.5) | 37 (17.5) |  |
| **Employment, n (%)** |  |  | 0.029 |  |  | 0.899 |
| Retired | 212 (64.8) | 103 (75.7) |  | 146 (67.0) | 128 (69.0) |  |
| Still working | 91 (27.8) | 22 (16.2) |  | 55 (25.2) | 52 (23.7) |  |
| Unemployed | 24 (7.3) | 11 (8.1) |  | 17 (7.8) | 18 (7.3) |  |
| **First-degree family history of PCa, n (%)** |  |  | 0.107 |  |  | 0.328 |
| No | 249 (76.2) | 112 (83.0) |  | 166 (76.2) | 195 (80.7) |  |
| Yes | 78 (23.8) | 23 (17.0) |  | 52 (23.8) | 49 (20.1) |  |
| Unknown | - | 1 |  | - | 1 |  |
| **Comorbilities, n (%)** |  |  | 0.046 |  |  | 0.001 |
| 0–2 | 310 (94.8) | 97 (89.7) |  | 212 (97.2) | 220 (89.8) |  |
| ≥3 | 17 (5.2) | 11 (10.3) |  | 6 (2.8) | 21 (10.2) |  |
| **BMI, n (%)** |  |  | 0.045 |  |  | 0.945 |
| Normal weight | 66 (20.2) | 24 (17.7) |  | 46 (21.1) | 49 (20.0) |  |
| Overweight | 174 (53.2) | 60 (44.1) |  | 112 (51.4) | 126 (51.4) |  |
| Obesity | 87 (26.6) | 52 (38.2) |  | 60 (27.5) | 70 (28.6) |  |
| **Smoking status, n (%)** |  |  | 0.315 |  |  | 0.144 |
| Never smoker | 89 (27.2) | 28 (20.6) |  | 51 (23.4) | 66 (26.9) |  |
| Former smoker | 173 (52.9) | 77 (56.6) |  | 128 (58.7) | 122 (49.8) |  |
| Current smoker | 65 (19.9) | 31 (22.8) |  | 39 (17.9) | 57 (23.3) |  |
| **Sedentary lifestyle, n (%)** |  |  | <0.001 |  |  | 0.572 |
| T1 (≤6 h/day) | 167 (51.1) | 32 (23.5) |  | 95 (43.6) | 104 (42.5) |  |
| T2 (6–9 h/day) | 90 (27.5) | 52 (38.2) |  | 62 (28.4) | 80 (30.6) |  |
| T3 (>9 h/day) | 70 (21.4) | 52 (38.2) |  | 61 (28.0) | 61 (24.9) |  |
| **Mediterranean diet pattern, mean (SD)** | 34.9 (3.8) | 34.8 (3.9) | 0.852 | 35.4 (3.6) | 34.4 (4.0) | 0.009 |
| **Urinary symptoms, n (%)** |  |  | 0.001 |  |  | <0.001 |
| Without | 101 (30.9) | 26 (19.1) |  | 76 (34.9) | 51 (20.8) |  |
| Mild | 125 (38.2) | 55 (40.4) |  | 83 (38.1) | 97 (39.6) |  |
| Moderate | 87 (26.6) | 36 (26.5) |  | 53 (24.3) | 70 (28.6) |  |
| Severe | 14 (4.3) | 19 (14.0) |  | 6 (2.7) | 27 (11.0) |  |
| **Tumor aggressiveness^a^, n (%)** |  |  | 0.138 |  |  | 0.901 |
| Low (ISUP 1–2) | 256 (78.3) | 97 (71.9) |  | 166 (76.1) | 187 (76.6) |  |
| High (ISUP 3–5) | 71 (21.7) | 38 (28.1) |  | 52 (23.9) | 57 (23.4) |  |
| **EAU risk groups, n (%)** |  |  | 0.007 |  |  | 0.520 |
| Localized PCa | 292 (89.3) | 109 (80.1) |  | 188 (86.2) | 213 (86.9) |  |
| Locally advanced PCa | 25 (7.6) | 14 (10.3) |  | 21 (9.6) | 18 (7.4) |  |
| Metastasic PCa | 10 (3.1) | 13 (9.6) |  | 9 (4.1) | 14 (5.7) |  |
| BMI, body mass index; EAU, European Association of Urology; PCa, Prostate Cancer; SD, standard deviation; SF-12 v2, Short-Form Health Survey version 2; T, Tercile.^a^One subject could not be categorized using ISUP classification as it was a neuroendocrine carcinoma. | | | | | | |
